# Outcome of Different Sequencing and Assembly Approaches on the Detection of Plasmids and Localization of Antimicrobial Resistance Genes in Commensal *Escherichia coli*

**DOI:** 10.3390/microorganisms9030598

**Published:** 2021-03-14

**Authors:** Katharina Juraschek, Maria Borowiak, Simon H. Tausch, Burkhard Malorny, Annemarie Käsbohrer, Saria Otani, Stefan Schwarz, Diana Meemken, Carlus Deneke, Jens Andre Hammerl

**Affiliations:** 1 Epidemiology, Zoonoses and Antimicrobial Resistance, German Federal Institute for Risk Assessment (BfR), Max-Dohrn Str. 8-10, 10589 Berlin, Germany; annemarie.kaesbohrer@bfr.bund.de; 2 Study Centre for Genome Sequencing and Analysis, German Federal Institute for Risk Assessment (BfR), Max-Dohrn Str. 8-10, 10589 Berlin, Germany; Maria.borowiak@bfr.bund.de (M.B.); simon.tausch@bfr.bund.de (S.H.T.); Burkhard.Malorny@bfr.bund.de (B.M.); Carlus.Deneke@bfr.bund.de (C.D.); 3 Unit for Veterinary Public Health and Epidemiology, University of Veterinary Medicine, Veterinaerplatz 1, 1210 Vienna, Austria; 4 DTU Food, National Food Institute, Technical University of Denmark, Kemitorvet, Building 204, 2800 Kgs Lyngby, Denmark; saot@food.dtu.dk; 5 Institute of Microbiology and Epizootics, Department of Veterinary Medicine, Freie Universität Berlin, 14163 Berlin, Germany; Stefan.Schwarz@fu-berlin.de; 6 Institute of Food Safety and Food Hygiene, Working Group Meat Hygiene, Department of Veterinary Medicine, Freie Universität Berlin, 14163 Berlin, Germany; diana.meemken@fu-berlin.de

**Keywords:** AMR, mobile genetic elements, *qnrS*, hybrid assembly, long-read sequencing, short-read sequencing

## Abstract

Antimicrobial resistance (AMR) is a major threat to public health worldwide. Currently, AMR typing changes from phenotypic testing to whole-genome sequence (WGS)-based detection of resistance determinants for a better understanding of the isolate diversity and elements involved in gene transmission (e.g., plasmids, bacteriophages, transposons). However, the use of WGS data in monitoring purposes requires suitable techniques, standardized parameters and approved guidelines for reliable AMR gene detection and prediction of their association with mobile genetic elements (plasmids). In this study, different sequencing and assembly strategies were tested for their suitability in AMR monitoring in *Escherichia coli* in the routines of the German National Reference Laboratory for Antimicrobial Resistances. To assess the outcomes of the different approaches, results from in silico predictions were compared with conventional phenotypic- and genotypic-typing data. With the focus on (fluoro)quinolone-resistant *E.* *coli*, five *qnrS*-positive isolates with multiple extrachromosomal elements were subjected to WGS with NextSeq (Illumina), PacBio (Pacific BioSciences) and ONT (Oxford Nanopore) for in depth characterization of the *qnrS1*-carrying plasmids. Raw reads from short- and long-read sequencing were assembled individually by Unicycler or Flye or a combination of both (hybrid assembly). The generated contigs were subjected to bioinformatics analysis. Based on the generated data, assembly of long-read sequences are error prone and can yield in a loss of small plasmid genomes. In contrast, short-read sequencing was shown to be insufficient for the prediction of a linkage of AMR genes (e.g., *qnrS1*) to specific plasmid sequences. Furthermore, short-read sequencing failed to detect certain duplications and was unsuitable for genome finishing. Overall, the hybrid assembly led to the most comprehensive typing results, especially in predicting associations of AMR genes and mobile genetic elements. Thus, the use of different sequencing technologies and hybrid assemblies currently represents the best approach for reliable AMR typing and risk assessment.

## 1. Introduction

Antimicrobial resistance (AMR) in food- and livestock-associated bacteria can represent an important threat to public health and needs to be monitored [1,2]. Currently, the mandated AMR monitoring in European countries generates broad datasets on minimal inhibitory concentrations (MICs) of country- and matrix-specific isolates against selected antimicrobial agents [3]. For this, commensal *Escherichia* (*E.*) *coli* were chosen as indicator organisms, as they belong to the common intestinal microbiota of livestock and thus reflect trends in the development of antimicrobial resistances associated with the lifestyle of animals [4]. Up to now, the use of phenotypic data represents the gold standard for AMR monitoring [5]. However, due to the broad diversity of determinants associated with decreased susceptibilities of isolates against specific antimicrobial classes, whole-genome sequencing (WGS) provides deeper insight into the genetic basis of antimicrobial resistances, possible routes of transmissions and important clonal lineages, which are useful for risk assessment [6]. Therefore, the European Food Safety Authority (EFSA) strongly advocates the implementation of WGS into the AMR monitoring of the national reference laboratories [7]. A WGS-based monitoring will prospectively provide a uniform basis for the identification of dissemination paths of genetic elements, supporting the fight against resistance development in livestock-associated and foodborne commensals and pathogens [8]. Furthermore, sequencing data will be available for retrospective analyses of novel resistance or virulence determinants [9]. However, to ensure the high quality WGS data, the prevailing techniques need to be standardized, and minimum quality parameters for the multisite use of datasets need to be specified. For reliable AMR prediction and correct plasmid detection, high throughput sequencing with high accuracy and reasonable cost is required. The selection of sequencing and assembly approaches can significantly influence the results of resistance gene detection and localization [10]. As plasmids are commonly implicated in the dissemination of AMR, it is important to correctly determine whether resistance genes are fixed on the chromosome or located on mobile genetic elements (MGEs) [11].

WGS evolved from first generation sequencing to high throughput next generation sequencing (NGS) up to long-read, real-time sequencing, known as third generation sequencing [9]. Second generation sequencing (SGS) platforms are known for relatively low costs, high throughput and shorter read lengths and are usually the first choice in routine diagnostics [12,13]. However, second generation sequencing has its limitations, as it usually includes a PCR amplification step, which can introduce a bias and nucleotide alterations during DNA synthesis. In addition, its short-read lengths make it unfavorable for some biological tasks [14,15]. Especially mobile genetic elements can be complex in their composition, making it difficult to determine them correctly [16]. Single-molecule real-time (SMRT) sequencing (Pacific Bioscience: PacBio) and Nanopore sequencing (Oxford Nanopore Technologies: ONT) are the dominant methods of third generation sequencing [17]. Although both techniques offer longer reads compared to SGS, their drawbacks include a lower throughput and a significantly higher error rate [14], making them rather disadvantageous for routine and outbreak diagnostics. According to the strengths and weaknesses, a combination of short- and long-read sequencing seems to be promising for the determination of complex genomic regions [14] or complete plasmid sequences.

Due to the importance of (fluoro)quinolones in human medicine [18], the steadily increasing number of *E. coli* developing resistances against substances of these classes represents an emerging risk to public health [19]. Decreased susceptibility against (fluoro)quinolones is based on diverse genetic determinants, as chromosomal alterations of the DNA gyrase/topoisomerase genes and plasmid-acquired determinants lead to modified aminoglycoside acetyltransferases (AAC(6′)-Ib-cr) [20], specific efflux pumps (QepA, OqxAB) [21] and pentapeptide repeat proteins (Qnr). However, the acquisition of some plasmid-associated genes in *E. coli* is not necessarily linked to the development of a phenotypical resistance, according to epidemiological or clinical interpretation guidelines [22]. Thus, determinants affecting the susceptibility of isolates to (fluoro)quinolones might spread unnoticed in *Enterobacteriaceae* by horizontal gene transfer. It has been hypothesized that some of the acquired determinants can also force chromosomal alterations in the DNA gyrase/topoisomerase, ultimately leading to strong phenotypic resistance of the isolates [23]. Thus, there is an urgent need for the characterization of transmissible (fluoro)quinolone resistance determinants to get deeper insights into the diversity of their hosting plasmids, the potential transmission pathways and their impact on resistance development.

In this study, the short- and long-read sequencing data were compared with results from molecular (i.e., pulsed-field gel electrophoresis (PFGE) macro-restriction and plasmid profiling) and microbiological analyses (i.e., minimum inhibitory concentration (MIC) determination) to investigate the impact of different sequencing and assembly strategies on the detection of resistance genes and the characterization of plasmids. This study has a particular focus on the detection of *qnrS1*, as this gene represents the most frequently found transmissible determinant associated with (fluoro)quinolone resistance in German livestock and food [24]. However, considering all available sequencing approaches and assembly pipelines published thus far, this work does not aim to represent an exhaustive comparison of all methods. Nevertheless, the provided raw and assembled sequencing data can be used by other groups to assess and evaluate their established assembly and annotation pipelines. The generated data and analysis of this study will support (i) the improvement of AMR monitoring for commensal *E. coli*, by implementing WGS as a gold standard for AMR prediction and (ii) an improved determination of MGEs associated with AMR gene prediction in the terms of risk assessments.

## 2. Materials and Methods

### 2.1. Selection of Bacterial Isolates

Five pre-selected *qnrS1*-positive *E. coli* from different sources with individual resistance profiles and multiple extrachromosomal elements were included in this study. These isolates originated from the German annual AMR monitoring program in 2016/2017 and were obtained from different sources, including poultry (*n* = 2), pig cecum (*n* = 1), calf cecum (*n* = 1) and bovine meat (*n* = 1). The five isolates represent common plasmid types of (fluoro)quinolone-resistant and *qnrS1*-positive *E. coli* from German livestock and food.

#### 2.1.1. Antimicrobial Susceptibility Testing

For determination of the minimum inhibitory concentration (MIC), the isolates were subjected to broth microdilution according to EUCAST (European Committee on Antimicrobial Susceptibility Testing) recommendations on a universal European antimicrobial test panel (Sensititre™, TREK Diagnostic Systems, East Grinstead, UK). The tested antimicrobial agents (Supplement S5) were used according to the European Commission Implementing Decision No. 2013/652/EU [25] for the monitoring and reporting of antimicrobial resistance in zoonotic and commensal bacteria [25]. The *E. coli* strain ATCC 25922 was included in MIC determination as quality control. MIC values were interpreted according to EUCAST epidemiological cut-off (ECOFF) values [26].

#### 2.1.2. PFGE Profiling and Plasmid Prediction

Pulsed-field gel electrophoresis (PFGE) was performed according to the PulsNet protocol [27]. Macro-restriction of genomic DNA was conducted using the restriction endonuclease XbaI (Thermo Fisher Scientific, Darmstadt, Germany). In addition, S1 nuclease (Thermo Fisher Scientific) PFGE was used to determine the presence and size of plasmids. Enzymatically treated agarose plugs were embedded in 1% agarose gels and separated in a CHEF-DR III system (Bio-Rad Laboratories, Madrid, Spain). For size determination, the *Salmonella* Braenderup strain H9812 was used.

For the detection of plasmids <20 kb, extrachromosomal DNA (pDNA) was isolated with the CosMCprep “Mini prep of plasmids” kit (Beckman, Krefeld, Germany) according to the manufacturer’s protocol. Plasmid visualization was performed in 0.8% agarose gels (Biozym Gold Agarose, Biozym, Vienna, Austria) separated for 1.5 h at 90 V.

For localization of *qnrS1*, Southern blotting and DNA-DNA hybridization of S1-PFGE gels were conducted using a digoxigenin-labelled (Roche Diagnostics, Mannheim-Penzberg, Germany) *qnrS1* probe, as previously described [28].

#### 2.1.3. Gene Prediction with PCR

For estimation of resistance genes, PCR-based detection of *qnrS1* was conducted on a Bio-Rad CFX system, as previously described [29]. For detecting *bla*_TEM_, primers and conditions were used as described elsewhere [30].

#### 2.1.4. In Vitro Filter Mating Experiments

For in vitro filter mating experiments, the sodium azide tolerant *E. coli* strain J53 was used as a recipient. All strains were grown in LB (lysogeny broth) to an OD_600_ of 0.8. A 500 μL aliquot of the donor was mixed with 1 mL of the recipient bacteria. The bacterial suspension was centrifuged at 5000× *g* for 10 min and the supernatant was discarded. Sedimented bacteria were applied onto a Millipore filter membrane (0.45 μm pore-size) on LB agar. After an incubation of 12–16 h at 37 °C, bacteria were removed from the filter by suspension in 5 mL of 0.7% (*w/v*) saline solution. An aliquot of 100 μL was applied onto LB agar supplemented with nalidixic acid (0.15 mg/L) and sodium azide (100 mg/L). The plates were incubated at 37 °C for 20–24 h. Upcoming colonies were stored in glycerol at −80 °C and subjected to S1-PFGE for determination of the plasmid transfer.

### 2.2. Genomic DNA Extraction for Whole-Genome Sequencing (WGS)

A single colony was cultivated in 12 mL LB and incubated for 14–16 h at 37 °C. After incubation, the culture was centrifuged at 4500× *g* for 10 min. The pellet was resuspended in 300 µL phosphate-buffered saline (PBS), and 10 mL of the extraction buffer (100 mM NaCl, 10 mM Tris-Cl, pH 8.0, 25 mM EDTA, pH 8.0, 0.5% (*w/v*) SDS, 20 µg/mL RNase A (Qiagen, Hilden, Germany)) (modified from Sambrook & Russell, 2001 [31]) was added. The solution was mixed and incubated for 1.5 h at 37 °C. Thereafter, Proteinase K (20 mg/mL; Qiagen) was added, and the mixture was incubated for another 1.5 h at 50 °C. The lysate was then separated into two 15 mL tubes (Thermo Fisher Scientific), and 5 mL of saturated phenol (Sigma-Aldrich, Taufkirchen, Germany) was added to each tube. The mixture was rotated for 20 min on a PTR-35 tube rotator (Grant-instruments, Cambridgeshire, Great-Britain) at 20 rpm. Afterwards, it was centrifuged at 4500× *g*. The aqueous phase was transferred into a new 15 mL tube. Again, 2.5 mL saturated phenol (~73%) and 2.5 mL phenol-chloroform-isoamyl alcohol (25:24:1) (Sigma-Aldrich) were added. The tubes were rotated at 20 rpm on the tube rotator and centrifuged at 4500× *g*. The clean aqueous phases of each tube were transferred into a new 15 mL tube, 5 M ammonium acetate (Sigma-Aldrich) was added, followed by 25 mL of ice-cold ethanol (>99.5%, Sigma-Aldrich). After 5 min incubation at room temperature, clouds of DNA threads were collected with an inoculation loop and transferred into 1 mL of 70% ethanol in a 2 mL tube. The tube was centrifuged for 10 min at 10,000× *g* and washed with 1 mL 70% ethanol (*v/v*). The obtained pellet was dissolved in 500 µL elution buffer and incubated overnight at 5 °C. The extracted high molecular weight DNA was stored at 5 °C until further use.

### 2.3. Whole-Genome Sequencing

To ensure the use of high-quality DNA for sequencing on the different platforms, quantification with the Qubit fluorometer and quality assessment with the fragment analyzer, according to their protocols, was conducted (Supplement S1). For short-read sequencing on the Illumina NextSeq 500, DNA libraries prepared with the Nextera DNA Flex Library Preparation Kit (Illumina) according to the manufacturer’s protocol were used. NextSeq sequencing was performed in 2 × 151 cycles with the Illumina NextSeq 500/550 Mid Output Kit v2.5 (300 Cycles) [32]. For long-read sequencing with the PacBio SMRT sequencing technology, DNA library preparation was conducted according to the standard manufacturer’s conditions in the protocol “Preparing Multiplexed Microbial Libraries Using SMRTbell Express Template Prep Kit 2.0” (Part Number 101-696-100, Version 02, February 2019). This protocol includes a size selection step, conducted with AMPure beats, removing SMRTbell templates < 3 kb. For long-read sequencing with the Oxford Nanopore technology (ONT), sequencing libraries were prepared using the Rapid Barcoding Kit (SQK-RBK004, ONT) and sequenced on an ONT MinION sequencer connected to an ONT MinIT v19.12.5 device (including Guppy base caller v3.2.10) using a FLO-MIN106 R9 flow cell.

### 2.4. De Novo Assembly Strategies and Genome Characterization

After sequencing, all short-reads were pre-processed and filtered with fastp under default parameters [33]. For the assembly of NextSeq short-read sequencing data, Unicycler v0.4.8 [34] was used. Reads generated with PacBio and MinION (ONT) were assembled using Flye v2.8.1 [35]. Hybrid assemblies were generated with Unicycler v0.4.8 under default parameters.

Assembled contigs were analyzed with abricate Version 1.0.1 [36,37,38,39,40,41,42,43] and platon 1.4.0 [42,44,45]. Results from abricate and platon were used to locate AMR genes. Annotation of plasmids was conducted with the PATRIC RASTk-enabled Genome Annotation service [46]. Visualization of *qnrS*-carrying plasmids was done with Unicycler assembled PacBio reads with the Blast Ring Image Generator (BRIG; v0.95) [47]. The transmissibility of plasmid genomes was assessed using the mob-suite tool [48]. The closest related plasmid was detected with a blastn search [49].

### 2.5. Accession Numbers

The complete datasets (raw reads) from different sequencing approaches were deposited in GenBank under the BioProject ID PRJNA589028. Accession numbers of the individual datasets are given in Supplement S2. Genome assemblies of the individual datasets are given in Supplement S7.

## 3. Results and Discussion

### 3.1. Impact of Different Long- and Short-Read Sequencing Approaches

To estimate the impact of different sequencing and assembly strategies for reliable in silico prediction of *qnrS1*-carrying plasmids in *E. coli*, five isolates representing multiple extrachromosomal elements and *qnrS1*-carrying plasmids of different size ranges (Table 1) were chosen for in depth characterization. De novo assembly from the data of NextSeq, ONT and PacBio sequencing (Supplement S7) varied according to the expectations for the outcome of short- and long-read sequencing. Overall, all sequencing data met the recommended requirements for accuracy, coverage, read length and the range of read counts (data not shown) for Illumina, Oxford Nanopore and Pacific Biosciences, respectively. This suggests the use of high-quality sequencing runs for bioinformatics analyses. In addition to the recommended quality parameter, the use of specific extraction methods can influence results obtained by whole-genome sequencing. However, the impact of different extraction systems seemed to have a lesser influence on the outcome of WGS [50].

We analyzed how the different sequencing and assembly approaches per sample affect the outcome of in silico typing for the number and size of predicted plasmids and the AMR associated with them. First, the different approaches were assessed for their suitability for bacterial chromosome finishing (Table 2). By using NextSeq data, none of the chromosomes of any isolate could be finished. In contrast, ONT data alone or in combination with NextSeq sequences allowed closing of the longest contig of every sample. Chromosome finishing using PacBio sequences failed for two of the five samples. However, the Unicycler-PacBio/-NextSeq hybrid assembly resulted in closed chromosomes for four isolates. Although long-read assembly (Flye) approaches frequently generated finished chromosomes, the result of the hybrid approach led to slightly longer closed chromosomal contigs with a higher accuracy, as wrongly predicted deletions were corrected. This leads to the presumption that the addition of the short-reads in the hybrid approach can replenish the chromosome with data otherwise missed in the long-read-only approach, although stated as already closed. Thus, our data are in good agreement with prevailing reports indicating that WGS approaches aiming for a full reconstruction of all genomic elements of an isolate will benefit from long-read or hybrid sequencing data [8,51,52].

While short-read sequencing applications yielded a high sequence accuracy, the technology is known to be insufficient for closing whole genome structures. Reliable estimation of MGEs within an organism might be challenging without additional information [14,53,54,55,56]. In contrast, long-read sequencing applications are more reliable in detection and closing of, e.g., extrachromosomal elements, but are assumed to be error prone for the prediction of specific genes under some circumstances [34,57,58].

### 3.2. Small Plasmids Are Difficult to Detect

**Figure 1:**
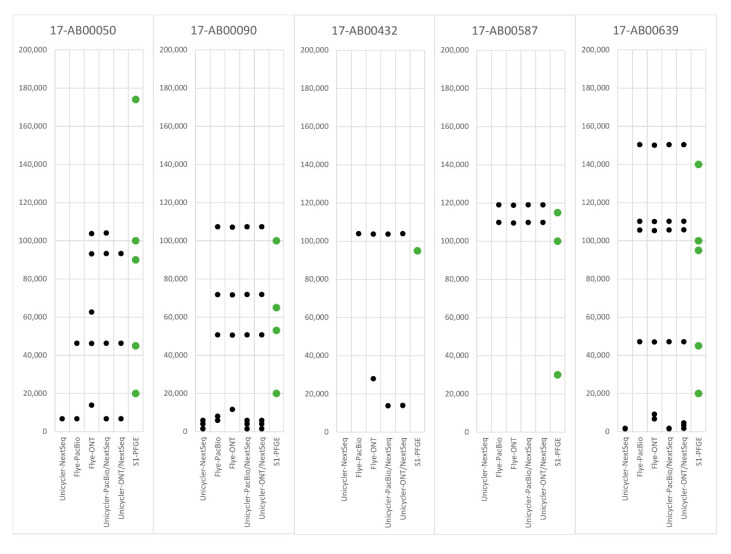
Distribution of plasmid size, detected with different sequencing and assembly approaches. A black dot represents a determined closed plasmid at the respective size for the given method. A green dot represents the size of the plasmid, detected in the laboratory with S1-PFGE.

To get an overview on transmissible extrachromosomal elements, the number and size of plasmids detected by S1-PFGE were compared to circularized contigs per sample identified in silico (Table 1, Figure 1, Supplement S3, Supplement S4). As S1-PFGE is unsuitable for reliable size estimation of small plasmids (<20 kb), agarose gel electrophoresis of plasmid DNA was conducted for confirming their presence (data not shown).

For isolate 17-AB00050, all assembly methods detected a 6.7 kb plasmid, which was assigned to the Col156 Inc-group. The MinION long-read-only approach (Flye-ONT) generated a genome of 13 kb, which is represented by a duplication of the 6.7 kb Col156 plasmid. Except for the Col156 plasmid, short-read (NextSeq) sequencing was insufficient to yield any further closed plasmid genomes for this isolate. All long-read-only (Flye-ONT, Flye-PacBio) and hybrid approaches (Unicycler-PacBio/Nextseq, Unicycler ONT/NextSeq) correctly recognized the 46 kb IncX3 plasmid. The Flye assembler generated a 62 kb plasmid that could be linked to the p0111 Inc-group. However, no other assembly method identified this plasmid, and the S1-PFGE also showed no evidence for its biological presence (Supplement S3). Both hybrid approaches, as well as Flye-assembled ONT and PacBio sequences, resulted in a 93 kb plasmid. However, no method was able to link this plasmid to a known Inc group. Only Flye-ONT and Flye-PacBio as well as the Unicycler-PacBio hybrid assembly led to the detection of the ~103 kb IncFIB plasmid (Figure 1). Finally, no in silico based prediction was able to detect the 174 kb plasmid, recognized by the S1-PFGE analysis of this isolate (Table 1, Supplement S3). For *E. coli* 17-AB00090, all long-read and hybrid assembly approaches reliably detected a 50 kb IncX1, a 71 kb IncFII and a 107 kb IncI-α plasmid. Furthermore, both hybrid assembly approaches recognized the same small plasmids (a 1551 bp Col(MG828), a 4018 bp ColRNAI and a 5873 bp ColRNAI plasmid) as the Unicycler-NextSeq assembly. Both Flye-ONT and Flye-PacBio resulted in double-sized plasmid genomes of 8 and 11 kb, where the complete sequence and the Inc ColRNAI sequence were duplicated.

All long-read and hybrid assembly approaches recognized the 103 kb IncY plasmid within the strain 17-AB00432. Furthermore, the hybrid approaches as well as Flye assembled ONT and PacBio sequences recognized another 13 kb IncR plasmid, which was not observed by S1-PFGE (Supplement S3). However, Flye again generated a doubled sized plasmid with a full duplication of the sequence, including the IncR marker. For *E. coli* 17-AB00587, all Flye and hybrid assembly approaches were able to recognize a 109 kb IncI-α and a 119 kb IncFIB plasmid. However, no assembly approach detected the 30 kb plasmid, which was reliably detectable by S1-PFGE (Supplement S3).

For *E. coli* 17-AB00639, all Flye and hybrid assemblies identified a 150 kb IncFII plasmid and a 105 kb IncI-α plasmid. In these assemblies, a 110 kb plasmid was further detected but could not be linked to a known incompatibility group. A 47 kb plasmid was also detected by these assembly methods. However, both hybrid assemblies assigned an IncX1, while both Flye approaches further assigned an IncX3 marker to this sequence. The NextSeq assembly and the hybrid assemblies detected a 1552 bp Col(MG828) and a 1748 bp CoplVC plasmid. Furthermore, the Unicycler-ONT assembly yielded a 3374 bp plasmid, which could not be linked to any known Inc group, and a 4593 bp ColRNAI plasmid. These plasmids were found in duplicated size for the Flye output. Overall, all plasmids of this isolate could be reliably detected by S1-PFGE (Supplement S3), while the sizes between in vitro and in silico investigations differed (Figure 1).

Overall, the NextSeq approach resulted in the highest discrepancy for plasmid prediction. Although it always detected circularized plasmids below 10 kb, when they were detected by gel electrophoresis, the NextSeq assembly did not result in closing of any larger plasmid. However, the detection of the Inc group with NextSeq sequencing was reliable and is therefore useful as a reference. In summary, the hybrid assembly with Unicycler was assessed as the most reliable approach to detect the number of extrachromosomal DNA correctly, linking them to a certain Inc group and closing these elements. The hybrid approach combines the benefits of short- and long-read sequencing for the detection of small plasmids and the genome finishing of large plasmids and chromosomes. Furthermore, Flye-ONT and Flye-PacBio assemblies sometimes resulted in the detection of plasmids that were not detectable by PFGE. In addition, duplicated Inc sequences or duplication of the complete plasmid sequence was observed, potentially leading to misinterpretations. It is known that long-read assembly can exclude short extrachromosomal DNA elements [34,59] due to size-selection or bead clean-up steps. This can lead to an exclusion of small plasmids, e.g., harbouring resistance and virulence genes [10], which might affect the assessment of the isolate. As plasmids are important vectors for transmitting resistance determinants, the correct determination of their presence is important [60,61,62]. It is of high importance for a correct risk assessment to recognize whether a gene is located on a MGE or fixed on the chromosome [63,64]. Based on our data, we propose the use of long-read sequencing for chromosome finishing; the use of PacBio or ONT did not affect the outcome. However, the data needs to be handled with care during estimation of the exact number of chromosomal elements in a sample. Hybrid assembly represents the most useful and powerful tool for plasmid counting as well as for reliable size and Inc-group prediction of smaller and larger plasmids. Besides methodological influences, the use of specific algorithms and pipelines also influences the detection of plasmid associated sequences [65]. While some of these are based only on the detection of plasmid replicon sequences [42], others use similarity and identity values for experimentally confirmed plasmid databases.

### 3.3. Hybrid Assembly Allows a Deep Insight into the Plasmid Structure

**Figure 2:**
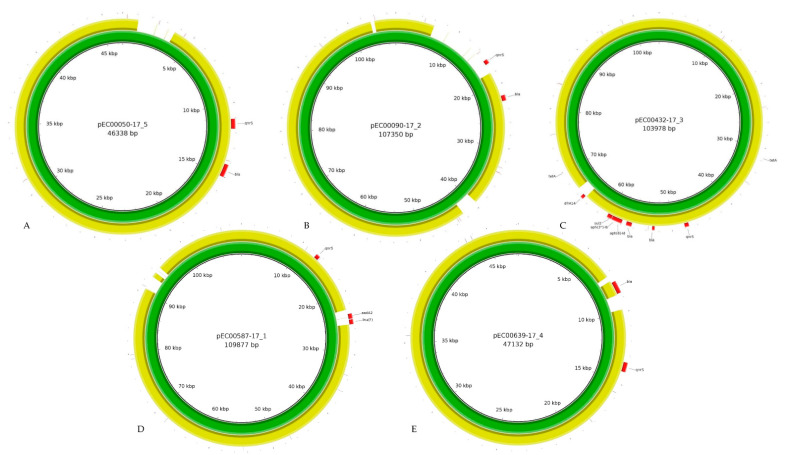
Plasmid composition of extrachromosomal elements carrying the *qnrS1* gene. (**A**): 17-AB00050 (**B**): 17-AB00090 (**C**): 17-AB00432 (**D**): 17-AB00587 (**E**): 17-AB00639. Illustrations were generated from hybrid assembly with BRIG (v0.95). Red elements represent the resistance genes. Green colored rings represent the isolates plasmid. Yellow colored rings represent the reference plasmid (annotation of plasmids are available in Supplement S6).

To determine the diversity of AMR-carrying plasmids and to understand the impact of resistance determinants and other plasmidal features (e.g., transposon sequences, transfer genes) for the spread of the genes, a deep knowledge of the composition of resistance plasmids is needed. With a focus on (fluoro)quinolone resistance, we aimed to dissect the *qnrS1*-carrying plasmids of the individual *E. coli* of livestock and food (Figure 2).

The *qnrS1* gene was detected with a 100% sequence identity in all isolates. However, for NextSeq sequences, the linkage of *qnrS1* to a plasmid incompatibility marker was only possible for 17-AB00050. In contrast, all datasets based on assemblies using long reads successfully led to a prediction of an incompatibility marker for *qnrS1*-carrying plasmids (Table 3). Furthermore, the use of NextSeq assemblies provided no evidence for the linkage of any further resistance gene to the *qnrS1*-carrying plasmid.

The *qnrS1*-carrying plasmid pEC00050-17_5 (isolate 17-AB00050) was 46.3 kb in size and belonged to the IncX3 group. Besides *qnrS1*, the plasmid also carried the Extended Spectrum β-Lactam (ESBL) resistance *bla*_SHV-12_, which was flanked by two IS*6* elements, while the *qnrS1* gene was associated with an IS*Kra4* element. Overall, pEC00050-17_5 was closely related (query coverage: 94%, identity: 99.95%) to the *Citrobacter freundii* plasmid pCF12 (accession number: MT441556.1). In contrast to pCF12, pEC00050-17_5 additionally carried a 3 kb sequence encoding the IS*Sso4* insertion sequence of the IS*21* family. When investigated with the in silico mob-suite tool, pEC00050-17_5 was predicted to be conjugative, as it identified the MOB_P_ relaxase type and the MPF_T_ type. Despite the in silico prediction, the plasmid was not transmitted by in vitro filter mating studies in *E. coli* J53.

The 107 kb *qnrS1*-carrying plasmid pEC00090-17_2 (*E. coli* 17-AB00090) was found to be related to the *Salmonella enterica* plasmid pCE-R2-11-0435_92 (query coverage: 86%, identity: 99.42%) (accession number: CP016520.1) recovered from retail chicken in Canada. pEC00090-17_2 belongs to the IncI-α group and carried a *bla*_TEM-1_ β-lactam resistance gene in close proximity to *qnrS1*. The transmissibility of the plasmid was shown by the in silico mob-suite tool as well as by in vitro filter mating studies. Comparative sequence analysis revealed that pEC00090-17_2 carried additional sequences (~15 kb) encoding transposases, DNA invertases, hypothetical proteins and *qnrS1*, which were absent in pCE-R2-11-0435_92. The presence of *qnrS1* on pEC00090-17_2 indicates an evolution step due to the acquisition of additional resistance markers. The new region included the IS*26* insertion sequence and the cn_6346_IS26 and cn_6346_IS26 composite transposon, all from the IS*6* family, as well as the transposon Tn2.

The *qnrS1*-carrying plasmid pEC00431-17_3 (*E. coli* 17-AB00432) exhibited an IncY incompatibility sequence and closely resembled (query coverage: 98%, identity: 99.98%) the *E. coli* plasmid tig00003056 (accession number: CP021681.1). pEC00431-17_3 exhibited a size of 103 kb and carried several resistance genes, including *tet*(A), *qnrS1*, *bla*_CTX-M-15_, *bla*_TEM-1_, *aph(6)-Id*, *aph(3″)-Ib*, *sul*2 and *dfrA2*, and might thus pose a risk in spreading multiple resistance genes. *bla*_CTX-M-15_ and *qnrS1* are in proximity to each other, located on an IS*Kpn19* insertion sequence of the IS*Kra4* family. As shown in Figure 2 (Supplement S6), pEC00431-17_3 differed from tig00003056 in the acquisition of an integrase as well as the trimethoprim resistance-mediating dihydrofolate reductase gene *dfrA14*, located on the cn_3458_IS26 composite transposon, belonging to the IS*6* family.

Bioinformatically, the 109 kb *qnrS1*-carrying plasmid pEC00587-17_1 (*E. coli* 17-AB00587) was assigned to the IncI-1-α group. Besides *qnrS1*, the plasmid exhibited *aadA2*, an *lnu*(F) resistance gene. Similar to pEQ2, *qnrS1* of pEC00587-17_1 was found to be located on the cn_4905_ISKpn19 composite transposon from the IS*Kra4* family. The closest relative of pEC00587-17_1 was pEQ2 (query coverage: 94%, identity: 100%), from an *E. coli* isolate recovered from pig feces in the UK. Hence, 4 kb were additionally located on pEC00587-17_1, which are represented by genes encoding hypothetical proteins and the resistance genes *aadA2* and *lnu*(F), located on the cn_4072_IS26 composite transposon of the IS*6* family. Plasmid pEQ2 was described as a fusion of pEQ1 and a *qnrS1*-carrying IncX1 plasmid, encoding replication, maintenance, and conjugative transfer [66]. Here, we noted another adaption by the acquisition of additional resistance genes. pEC00587-17_1 was shown to be transmissible by both in silico prediction using mob-suite and filter mating studies.

The 47 kb *qnrS1*-carrying IncX plasmid pEC00639-17_4 (isolate 17-AB00639) resembled the similar sized *E. coli* plasmid pNVI2422 (query coverage: 96%, identity: 99.99%), recovered from turkey meat in Norway. Nucleotide differences between the plasmids are based on the presence and absence of gene coding for hypothetical proteins. Besides *qnrS1*, pEC00639-17_4 also carried the β-lactam resistance *bla*_TEM-1_, which was flanked by an IS*26* insertion sequence belonging to the IS6 family. The adjacent *qnrS1* gene was located near the IS*Kpn19* insertion sequence belonging to the IS*Kra4* family. The in silico predicted plasmid transmissibility could be experimentally confirmed.

Except for the NextSeq approach, all other assemblies resulted in the same plasmid genome prediction for all five strains. In general, any linkage between *qnrS1*, the plasmid type and its associated characteristics (e.g., broad/narrow host plasmid type, mobilization, etc.) could only be made by using Flye-ONT or Flye-PacBio. Thus, we were able to detect certain insertion elements leading to the acquisition of, e.g., resistance determinants as well as other components. We were able to detect the *qnrS* element frequently in proximity to certain *bla* genes, as described previously [67]. Furthermore, we linked the presence of *qnrS* to the presence of the IS*26* and IS*Kra4* family. These elements had been recognized before as important for the transmission of *qnr* genes [68]. However, these important observations regarding the plasmid structure and *qnrS* characteristic are not possible using NextSeq sequencing alone.

### 3.4. Common Mistakes in Resistance Gene Detection and Phenotype Evaluation

For a reliable assessment of a resistance transfer probability within and beyond species, the understanding of the genetic determinant and the surrounding environment is essential. Thus, the *E. coli* isolates were screened for the presence of specific antimicrobial resistance genes (Table 4) from data derived in silico and compared to respective MIC data. Therewith, different sequencing and assembly strategies resulted in wrong prediction due to a disparity in gene assignment as well as a lack of information about duplicated genes. For isolate 17-AB00090, the Flye-ONT assembly resulted in detection of the β-lactamase gene *bla*_TEM-135_. Other assembly strategies revealed *bla*_TEM-1_ instead of *bla*_TEM-135_ at the same position. As both genes exhibit 99.8% nucleotide identity, misinterpretation will affect only the prediction of the gene variant. However, for other resistance genes, the prediction of the wrong variant could alter the in silico prediction of the phenotype. Thus, mistakes of this kind could lead to wrong conclusions [69]. One major drawback of using WGS-based antimicrobial resistance prediction is that only known genes associated with resistance development can be reliably interpreted. However, some machine-learning algorithms for reliable prediction of novel antimicrobial resistance determinants have been developed and successively optimized [70,71].

NextSeq sequencing detected all occurring resistance genes only once for every sample. In contrast, all long-read and hybrid assembly approaches resulted in multiple duplications of certain resistance genes (Table 4), which does not necessarily influence the in silico based prediction of the resistance phenotype, but leads to a different organization of the affected plasmids. Further dissection using NextSeq data showed a slightly higher sequencing depth of the respective resistance gene regions, indicating that a duplication of the genes might exist.

### 3.5. Evaluation of the Phenotype

We further assessed the reliability of the different sequencing and assembly approaches for accurate resistance phenotype prediction. Antimicrobial susceptibility testing (AST) of the individual isolates was conducted in triplicate (Supplement S5) and compared to the respective in silico outcome.

Obviously, the failure of detecting certain resistance genes can lead to discordance in estimating the right phenotype (Table 4). Sequencing of strain 17-AB00090 resulted in the detection of the *bla*_EC-18_ gene (Accession: A0A244BQ89), which led to the prediction of cephalosporin resistance. However, AST provided no evidence for a non-wildtype phenotype to cephalosporins. We made similar observations for the predicted macrolide resistance phenotype of 17-AB00587 and 17-AB000639, based on the presence of *mph*(A). As most *E. coli* isolates are intrinsically resistant to macrolides, a change in the macrolide resistance phenotype will not be detectable, regardless of the presence or absence of *mph*(A). This incorrect classification underlines the current difficulties of extrapolating from WGS data to resistance phenotypes [72,73].

Here, all sequence approaches resulted in congruent estimation of resistance genes. Besides the quality of the sequence data used, there are also other reasons that led to differences in the results when genotypes and phenotypes were compared. First, the observed phenotype is rarely traceable to only one single resistance gene. Most often, co-occurrence of different resistance genes can account for the same resistance property, or resistance phenotypes may result from complex gene networks that cannot be determined by occurrence of single genes [73]. Furthermore, some resistance genes do not confer resistance, but only slightly increase the MIC value for the respective antimicrobial agent [74,75]. Genotypic approaches can misinterpret gene silencing or generally only determine known resistance genes. This means that resistant isolates carrying a novel resistance gene or a mutation can be incorrectly classified as susceptible. Thus, in silico phenotype estimation remains a complex task, only solvable in mutual approaches of bioinformatics and laboratory work [9]. All currently used WGS-based methods are generally appropriate for reliable antimicrobial resistance prediction. Nevertheless, the use of long-read data alone can lead to a wrong prediction of individual chromosomal alterations involved in the development of antimicrobial resistances, i.e., to quinolones or rifampicin [73]. However, further sequencing quality parameters as well as the used of harmonized antimicrobial resistance databases will improve their comparability.

## 4. Conclusions

Long-read sequencing is an essential approach for reliable genome finishing. However, long-read assembly alone can lead to wrong annotations as well as to a loss of small plasmid genomes. Although long-read approaches are beneficial for building the scaffold of a genome, they do not fulfill all requirements for a thorough assessment, as information can be missed or errors can be incorporated. Despite short-read sequencing being currently the most popular way to investigate the genetic background, it is insufficient for certain purposes. In particular, when detecting and characterizing extrachromosomal plasmids, short reads alone did not allow the linkage to a plasmid marker as well as closing of the respective contigs. This sequencing even missed duplications of certain resistance genes. This makes a correct plasmid profiling, to be included in the assessment of antibiotic resistance dissemination, rather difficult. While short-read sequencing and assembling is reliable to some extent in gene detection and resistance phenotype estimation, it remains insufficient for drawing complex conclusions. Short- and long-read approaches both have pros and cons, depending on the purpose of use. However, when the aim was to investigate extrachromosomal structures like plasmids, hybrid assembly led to the most comprehensive results, as it led to more appropriate resistance gene and phenotype detection. In addition, it combined the information of large contigs and the information of smaller reads missed in the long-read-only assembly. However, the source of the long-read sequences, whether from PacBio or ONT, did not result in an extensive difference for the detection and characterization of extrachromosomal DNA.

Overall, we consider a hybrid assembly as a necessary approach for a detailed strain characterization, since it benefits from a thorough overview of various sized extrachromosomal DNA and correct resistance gene estimation. Overall, it will be worth extending the routine sequence diagnostic from short-read sequencing to additional long-read sequencing for a hybrid assembly approach, when a reference-grade complete bacterial genome is desired, or extrachromosomal structures need to be fully understood.

## Figures and Tables

**Table 1:** Overview on basic information of the *E. coli* isolates, including their antimicrobial resistance profile and the size of extrachromosomal elements.

Isolate ID	Matrix	Date of Isolation	AMR Profile	Sizes of Identified Plasmids ^+^
17-AB00050	cecum, broiler	22 November 2016	AMP, CEF, CIP, FOT, GEN, SMX, TAZ	174 kb; 100 kb; 90 kb; 45 kb *; <20.5 kb
17-AB00090	feces, turkey	14 December 2016	AMP, CIP, NAL, TET	100 kb *; 65 kb; 53 kb; <20.5 kb
17-AB00432	cecum, calf	21 February 2017	AMP, CEF, CIP, FOT, NAL, TAZ, TET, TMP, SMX	95 kb *
17-AB00587	meat, bovine	23 March 2017	AMP, CEF, CIP, FOT, TAZ	115 kb; 100 kb *; 30 kb
17-AB00639	cecum, pig	24 April 2017	AMP, CEF, CIP, FOT, GEN, SMX, TAZ, TMP	140 kb; 100 kb; 95 kb; 45 kb *; <20.5 kb

Abbreviations: AMP: ampicillin, AZI: azithromycin, CEF: cefepime, FOT: cefotaxime, FOX: cefoxitin, TAZ: ceftazidime, CHL: chloramphenicol, CIP: ciprofloxacin, COL: colistin, GEN: gentamicin, NAL: nalidixic acid, SMX: sulfamethoxazole, TET: tetracycline, TGC: tigecycline, TMP: trimethoprim; *: plasmid carrying *qnrS*, identified by S1-PFGE analysis; ^+^: data was obtained from S1-PFGE analysis and DNA-DNA hybridization.

**Table 2:** Characteristics of contigs detected with different sequencing and assembly strategies in five *E. coli* isolates. Circularized contigs are presented in bold.

	17-AB00050			17-AB00090			17-AB00432			17-AB00587			17-AB00639		
	Number of Contigs (Plasmidal Content Relative to Total Contig Length)	Number of Circular Contigs/All Contigs < 10 kb	Longest Contig [bp]	Number of Contigs (Plasmidal Content Relative to Total Contig Length)	Number of Circular Contigs/Contigs < 10 kb	Longest Contig [bp]	Number of Contigs (Plasmidal Content Relative to Total Contig Length)	Number of Circular Contigs/Contigs < 10 kb	Longest Contig [bp]	Number of Contigs (Plasmidal Content Relative to Total Contig Length)	Number of Circular Contigs/Contigs < 10 kb	Longest Contig [bp]	Number of Contigs (Plasmidal Content Relative to Total Contig Length)	Number of Circular Contigs/Contigs < 10 kb	Longest Contig [bp]
**Unicycler-NextSeq**	466 (5.5%)	2/2	341,211	186 (4.3%)	3/3	502,527	159 (1.6%)	0/0	314,433	201 (4.2%)	0/0	485,939	183 (7.6%)	2/2	340,946
**Flye-PacBio**	13 (4.6%)	4/1	4094,393	6 (4.6%)	6/2	**5004,742**	2 (2.1%)	2/0	**4,736,227**	3 (4.4%)	3/0	**4,938,765**	7 (7.9%)	4/1	3,693,252
**Flye-ONT**	8 (5.3%)	7/0	**5,524,427**	5 (4.6%)	5/0	**4,970,722**	3 (2.7%)	3/0	**4,728,917**	3 (4.4%)	3/0	**4,931,189**	7 (8.3%)	7/4	**4,756,147**
**Unicycler-PacBio/NextSeq**	27 (4.2%)	6/2	4,341,057	7 (4.6%)	7/3	**5,004,751**	3 (2.4%)	3/0	**4,736,229**	3 (4.4%)	3/0	**4,938,758**	13 (8.1%)	7/2	**4,763,387**
**Unicycler-ONT/NextSeq**	22 (4.0%)	6/2	**5,533,851**	7 (4.6%)	7/3	**5,004,751**	3 (2.4%)	3/0	**4,736,229**	3 (4.4%)	3/0	**4,938,758**	9 (8.2%)	9/2	**4,763,387**

**Table 3:** Characteristics of contigs detected with different sequencing and assembly strategies in five *E. coli* isolates harboring the *qnrS1* gene. Circularized contigs are presented in bold.

	17-AB00050			17-AB00090			17-AB00432			17-AB00587			17-AB00639		
	Contig Size [bp]	Plasmid Marker	Other AMR Genes	Contig Size [bp]	lPlasmid Marker	Other AMR Genes	Contig Size [bp]	Plasmid Marker	Other AMR Genes	Contig Size [bp]	Plasmid Marker	Other AMR Genes	Contig Size [bp]	Plasmid Marker	Other AMR Genes
**Unicycler-NextSeq**	42,601	IncX3	-	5348	-	-	13,373	-	-	1762	-	-	8821	-	-
**Flye-PacBio**	**46,338**	IncX3	*bla*_SHV-12_	**107,341**	IncI1_1_α	*bla*_TEM-1_	**103,789**	IncY	*tet*(A), *bla*_CTX_M-15_, *bla*_TEM-1_, *aph*(3″)-ib, *sul*2, *dfr*A14, *tet*(A), *aph*(6)-Id	**109,877**	IncI1_1α	*aad*A2, *lnu*(F)	**47,133**	IncX1, IncX3	*bla*_TEM-1_
**Flye-ONT**	**46,207**	IncX3	*bla*_SHV-12_	**107,104**	IncI1_1_α	*bla*_TEM-1_	**103,779**	IncY	*tet*(A), *bla*_CTX_M-15_, *bla*_TEM-1_, *aph*(3″)-ib, *sul*2, *dfr*A14, *tet*(A), *aph*(6)-Id	**118,872**	IncI1_1α	*aad*A2, *lnu*(F)	**46,996**	IncX1, IncX3	*bla*_TEM-1_
**Unicycler-PacBio/NextSeq**	**46,338**	IncX3	*bla*_SHV-12_	**107,350**	IncI1_1_α	*bla*_TEM-1_	**103,978**	IncY	*tet*(A), *bla*_CTX_M-15_, *bla*_TEM-1_, *aph*(3″)-ib, *sul*2, *dfr*A14, *tet*(A)	**109,877**	IncI1_1α	*aad*A2, *lnu*(F)	**47,132**	IncX1	*bla*_TEM-1_
**Unicycler-ONT/NextSeq**	**46,338**	IncX3	*bla*_SHV-12_	**107,350**	IncI1_1_ α	*bla*_TEM-1_	**103,975**	IncY	*tet*(A), *bla*_CTX_M-15_, *bla*_TEM-1_, *aph*(3″)-ib, *sul*2, *dfr*A14, *tet*(A), *aph*(6)-Id	**109,876**	IncI1_1α	*aad*A2, *lnu*(F)	**47,132**	IncX1	*bla*_TEM-1_
**PFGE determined size [bp]**	45,000			100,000			95,000			100,000			45,000		
**in vitro conjugational transfer**	no			yes			no			yes			yes		

Abbreviation: AMR, antimicrobial resistance.

**Table 4:** Antimicrobial resistance phenotype and resistance determinants predicted with various assembly and sequencing techniques in five *E. coli* isolates.

**17-AB00050**				
**Class of In Silico Type Phenotype**	**Subclass of In Silico Type Phenotype**	**Determined Resistance Gene(s)**	**Determined Phenotype**	**Class/Subclass of Determined Phenotype**
Quinolone	Quinolone	*qnrS1*	Ciprofloxacin	Fluoroquinolone
β-Lactam	Penicillin, Cephalosporin	*bla*_EC-8_, *bla*_SHV-12_	Ampicillin	β-Lactam
			Cefepime	Cephalosporin
			Cefotaxime	Cephalosporin
Aminoglycoside	Gentamicin	*aac(3)-VIa*	Gentamicin	Aminoglycoside
Sulfonamide	Sulfonamid	*sul1*	Sulphamethoxazole	Sulfonamide
Aminoglycoside	Streptomycin	*aadA1*	Not within the test panel	Not within the test panel
**17-AB00090**				
**Class of In Silico Type Phenotype**	**Subclass of In Silico Type Phenotype**	**Determined Resistance Gene(s)**	**Determined Phenotype**	**Class/Subclass of Determined Phenotype**
β-Lactam	Penicillin	*bla*_TEM-1_*, bla*_TEM-1_*^1^*, bla*_TEM-135_*^2^	Ampicillin	β-Lactam
β-Lactam	Penicillin, Cephalosporine	*bla*_EC-18_		
Quinolone	Quinolone	*qnrS1*	Ciprofloxacin	Fluoroquinolone
			Nalidixic acid	Quinolone
Tetracycline	Tetracycline	*tet*(A)	Tetracycline	Tetracycline
**17-AB00432**				
**Class of In Silico Type Phenotype**	**Subclass of In Silico Type Phenotype**	**Determined Resistance Gene(s)**	**Determined Phenotype**	**Class/Subclass of Determined Phenotype**
β-Lactam	Penicillin	*bla*_TEM-1_	Ampicillin	β-Lactam
β-Lactam	Penicillin, Cephalosporin	*bla*_CTX-M-15_, *bla*_EC_	Cefepime	Cephalosporin
			Cefotaxime	Cephalosporin
			Ceftazidime	Cephalosporin
Aminoglycoside	Kanamycin	*aph(3′)-Ia*	Not within the test panel	Not within the test panel
Quinolone	Quinolone	*qnrS1*	Ciprofloxacin	Fluoroquinolone
			Nalidixic acid	Quinolone
Aminoglycoside	Streptomycin	*aph(3″)-Ib*, aph(3″)-Ib*	Not within the test panel	Not within the test panel
		*aph(3′)-Ia,*		
		*aph(6)-Id*, aph(6)-Id*		
Sulfonamide	Sulfonamide	*sul*2*, sul*2*	Sulphamethoxazole	Sulfonamide
Tetracycline	Tetracycline	*tet*(A), *tet*(A)*^3^, *tet*(B)	Tetracycline	Tetracycline
**17-AB00587**				
**Class of In Silico Type Phenotype**	**Subclass of In Silico Type Phenotype**	**Determined Resistance Gene**	**Determined Phenotype**	**Class/Subclass of Determined Phenotype**
β-Lactam	Cephalosporin	*bla*_CTX-M-1_, *bla*_EC-15_	Cefepime	Cephalosporin
			Cefotaxime	Cephalosporin
			Ceftazidime	Cephalosporin
Lincosamide	Lincosamide	*lnu*(F)	Not within the test panel	Not within the test panel
Macrolide	Macrolide	*mph*(A)		
Quinolone	Fluoroquinolone	*qnrS1*	Ciprofloxacin	Fluoroquinolone
Aminoglycoside	Streptomycin	*aadA*2	Not within the test panel	Not within the test panel
**17-AB00639**				
**Class of In Silico Type Phenotype**	**Subclass of In Silico Type Phenotype**	**Determined Resistance Gene(s)**	**Determined Phenotype**	**Class/Subclass of Determined Phenotype**
β-Lactam	Penicillin	*bla*_TEM-1_	Ampicillin	β-Lactam
β-Lactam	Penicillin, Cephalosporin*^3^	*bla*_CTX-M-1_*^4^*, bla*_EC,_	Cefepime	Cephalosporin
		*bla*_TEM-1_*^4^, *bla*_TEM-1_	Cefotaxime	Cephalosporine
			Ceftazidime	Cephalosporine
Aminoglycoside	Gentamicin	*aac*(3)*-Iva*	Gentamicin	Aminoglycoside
Aminoglycoside	Hygromicin	*aph*(4)*-Ia*	Not within the test panel	Not within the test panel
Macrolide	Macrolide	*mph*(A)		
Quinolone	Fluoroquinolone	*qnrS1*	Ciprofloxacin	Fluoroquinolon
Aminoglycoside	Streptomycin	*aph(3″)-Ib*, aph(3″)-Ib*^4^,	Not within the test panel	Not within the test panel
		aph(6)-Id* ^5^		
Sulfonamide	Sulfonamide	*sul2*	Sulphamethoxazole	Sulfonamide
Diaminopyrimidine	Trimethoprim	*dfrA5*	Trimethoprim	Diaminopyrimidine

*^1^: duplication detected in all long-read and hybrid assemblies but not in NextSeq assemblies. *^2^: gene only determined with data Flye-ONT assemblies. *^3^: duplication detected in all long-read and hybrid assemblies but not in short-read-only assemblies. *^4^: detected in all long-read and hybrid assemblies but not in NextSeq assemblies. *^5^*:* not detected in short-read-only assemblies and Flye-ONT sequences.

## Data Availability

Sequencing data generated within this study are deposited in Genbank under the BioProject ID PRJNA589028.

## Supplementary Materials

The following are available online at https://www.mdpi.com/2076-2607/9/3/598/s1.
