# Supplementary material for: Outcome of Different Sequencing and Assembly Approaches on the Detection of Plasmids and Localization of Antimicrobial Resistance Genes in Commensal Escherichia coli
Source: Microorganisms. 2021 Mar 14;9(3):598. doi: 10.3390/microorganisms9030598 (PMC8000739; doi:10.3390/microorganisms9030598)
Supplement: Supplementary file 1 [file microorganisms-09-00598-s001.zip › Supplementary_Material_REVISION.pdf]

Supplementary Material

Supplement S1: Data evaluation

For evaluation of the genome assemblies, the quality assessment tool QUAST 5.0.2 was used. Thereby, we determined the number of contigs in various sizes, the size of the largest contig, the N50, and the number of circular contigs. Further, assembled contigs were analysed with mlst [1]. Assembled contigs were analysed for their resistance genes and plasmid markers with abricate Version 1.0.1 [2]. Databases were provided by abricate and downloaded on 09.24.20. These were the PlasmidFinder database [3] as well as the NCBI [4, 5] resistance database. For concordance, a minimal identity (ID) of 80 % and a minimal coverage of 80 % for estimation of plasmid markers and resistance genes was used as threshold. When used in conjunction with the NCBI resistance database, abricate provides for each investigated isolate its associated resistance phenotype.

Supplement S2: Accession Number

Supplementary Table S1: SRX-ID for different sequencing approaches within the Genbank BioProject ID PRJNA589028. SRS5635527 (17-AB00050), SRS5635528 (17-AB00090), SRS5635529 (17-AB00432), SRS5635530 (17-AB00587) and SRS5635526 (17-AB00639).

**Sample name and sequencing and assembly approach**	**SRX-ID**	**SRR-ID**
raw-reads MinION 17-AB00050	SRX9296037	SRR12828403
raw-reads MinION 17-AB00090	SRX9296038	SRR12828402
raw-reads MinION 17-AB00432	SRX9296039	SRR12828401
raw-reads MinION 17-AB00587	SRX9296040	SRR12828400
raw-reads MinION 17-AB00639	SRX9296041	SRR12828399
raw-reads PacBio 17-AB00050	SRX7222756	SRR10538960
raw-reads PacBio 17-AB00090	SRX7222757	SRR10538959
raw-reads PacBio 17-AB00432	SRX7222758	SRR10538958
raw-reads PacBio 17-AB00587	SRX7222759	SRR10538957
raw-reads PacBio 17-AB00639	SRX7222760	SRR10538956
Raw-reads Illumina NextSeq 17-AB00050	SRX7214604	SRR10530641
Raw-reads Illumina NextSeq 17-AB00090	SRX7214605	SRR10530640
Raw-reads Illumina NextSeq 17-AB00432	SRX7214606	SRR10530639
Raw-reads Illumina NextSeq 17-AB00587	SRX7214607	SRR10530638
Raw-reads Illumina NextSeq 17-AB00639	SRX7214608	SRR10530637

Supplement S3: (A) XbaI-PFGE profiles of *E. coli* isolates. S: *Salmonella* serovar Braenderup H9812, lane 1: 17-AB00050, lane 2: 17-AB00090, lane 3: 17-AB00432, lane 4: 17-AB00587 and lane 5: 17-AB00639. (B) S1-PFGE profile of the *E. coli* isolates and determination of the *qnrS1* gene location. S: *Salmonella* serovar Braenderup H9812, lane 1: 17-AB00050, lane 2: 17-AB00090, lane 3: 17-AB00432, lane 4: 17-AB00587 and lane 5: 17-AB00639. The location of the *qnrS1* was determined by DNA-DNA hybridization. Bands exhibiting positive hybridization signals using *qnrS1* as a probe are indicated by asterisks. (C) Results of the plasmid-profiling using CosMCprep "Mini prep of plasmids" kit (Beckman) extracted plasmid DNA on a 0.8 % agarose gel. S: DNA marker (1-10 kb) (Biozym, Oldendorf, Germany), lane 1: 17-AB00050, lane 2: 17-AB00090, lane 3: 17-AB00432, lane 4: 17-AB00587 and lane 5: 17-AB00639.

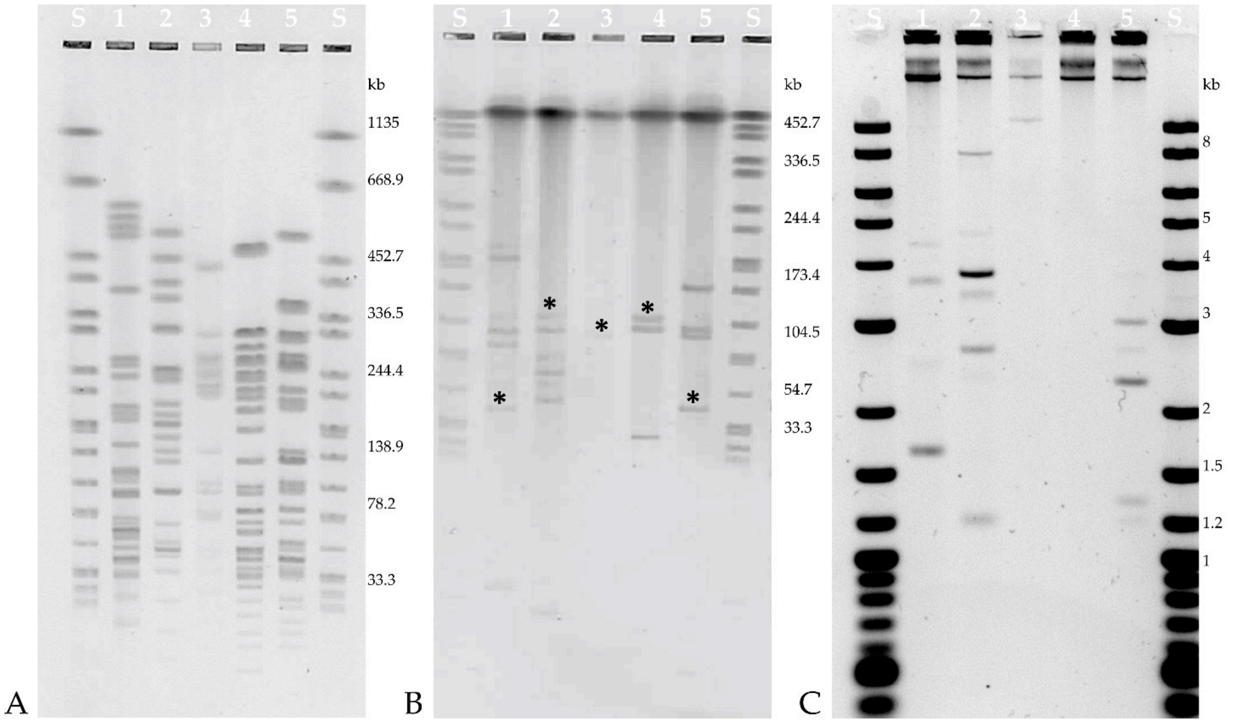

Supplement S4: Size, Inc group and respective resistance genes per closed plasmid detected through different sequencing and assembly approaches.

17-AB00050

S1-PFGE size (bp)	Unicycler-NextSeq	Flye-PacBio	Flye-ONT	Unicycler-PacBio/NextSeq	Unicycler-ONT/NextSeq
**< 20,000**	6,789<br>Col156<br>-	6,795<br>Col156<br>-	13,911<br>Col156, Col156<br>-	6,789<br>Col156<br>-	6,789<br>Col156<br>-
**45,000**	-<br>-<br>-	46,338<br>IncX3<br>*qnrS1*, *bla*SHV-12	46,207<br>IncX3<br>*qnrS1*, *bla*SHV-12	46,338<br>IncX3<br>*qnrS1*, *bla*SHV-12	46,338<br>IncX3<br>*qnrS1*, *bla*SHV-12
	-<br>-<br>-	-<br>-<br>-	62,702<br>p0111<br>-	-<br>-<br>-	-<br>-<br>-
**90,000**	-<br>-<br>-	-<br>-<br>-	93,200<br>-<br>-	93,318<br>-<br>-	93,318<br>-<br>-
**100,000**	-<br>-<br>-	-<br>-<br>-	103,735<br>IncFIB(AP001918)<br>*aad*A1, *aac*(3)-Via, *sul*1	104,102<br>IncFIB(AP001918)<br>*aad*A1, *aac*(3)-Via, *sul*1	-<br>-<br>-
**174,000**	-	-	-	-	-

17-AB00090

S1-PFGE size (bp)	Unicycler-NextSeq	Flye-PacBio	Flye-ONT	Unicycler-PacBio/ NextSeq	Unicycler-ONT/ NextSeq
**< 20,000**	1,551<br>Col(MG828)<br>-	-<br>-<br>-	-<br>-<br>-	1,551<br>Col(MG828)<br>-	1,551<br>Col(MG828)<br>-
	4,018<br>ColRNAI<br>-	-<br>-<br>-	-<br>-<br>-	4,018<br>ColRNAI<br>-	4,018<br>ColRNAI<br>-
	5,873<br>ColRNAI<br>-	5,873<br>ColRNAI<br>-	-<br>-<br>-	5,873<br>ColRNAI<br>-	5,873<br>ColRNAI<br>-
	-<br>-<br>-	8,036<br>ColRNAI, ColRNAI<br>-	11,689<br>ColRNAI, ColRNAI<br>-	-<br>-<br>-	-<br>-<br>-
**53,000**	-<br>-<br>-	50,698<br>IncX1<br>*tet*(A), *bla*$_{TEM-1}$	50,557<br>IncX1<br>*tet*(A), *bla*$_{TEM-135}$	50,699<br>IncX1<br>*tet*(A), *bla*$_{TEM-1}$	50,699<br>IncX1<br>*tet*(A), *bla*$_{TEM-1}$
**65,000**	-<br>-<br>-	71,840<br>IncFII(pHN7A8) pHN7A8<br>*bla*$_{TEM-1}$	71,683<br>IncFII(pHN7A8) pHN7A8<br>*bla*$_{TEM-1}$	71,850<br>IncFII(pHN7A8) pHN7A8<br>*bla*$_{TEM-1}$	71,850<br>IncFII(pHN7A8) pHN7A8<br>*bla*$_{TEM-1}$
**100,000**	-<br>-<br>-	107,341<br>IncI1_α<br>*bla*$_{TEM-1}$, *qnrS1*	107,104<br>IncI1_α<br>*bla*$_{TEM-1}$, *qnrS1*	107,350<br>IncI1_α<br>*bla*$_{TEM-1}$, *qnrS1*	107,350<br>IncI1_α<br>*bla*$_{TEM-1}$, *qnrS1*

17-AB00432

S1-PFGE size (bp)	Unicycler-NextSeq	Flye-PacBio	Flye-ONT	Unicycler-PacBio/ NextSeq	Unicycler-ONT/ NextSeq
	-<br>-<br>-	-<br>-<br>-	27,999<br>IncR_1, IncR_1<br>-	13,842<br>IncR_1<br>-	14,020<br>IncR_1<br>-
**95,000**	-<br>-<br>-	103,978<br>IncY<br>*tet*(A), *tet*(A), *dfrA*14, *sul*2, *aph*(3'')-Ib, *aph*(6)-Id, *bla*$_{\text{TEM-1}}$, *bla*$_{\text{CTX-M-15}}$, *qnrS1*	103,779<br>IncY<br>*tet*(A), *tet*(A), *dfrA*14, *sul*2, *aph*(3'')-Ib, *aph*(6)-Id, *bla*$_{\text{TEM-1}}$, *bla*$_{\text{CTX-M-15}}$, *qnrS1*	103,789<br>IncY<br>*tet*(A), *tet*(A), *dfrA*14, *sul*2, *aph*(3'')-Ib, *aph*(6)-Id, *bla*$_{\text{TEM-1}}$, *bla*$_{\text{CTX-M-15}}$, *qnrS1*	103,975<br>IncY<br>*tet*(A), *qnrS1*, *bla*$_{\text{CTX-M-15}}$, *blaTEM-1*, *aph(6)-Id*, *aph(3'')-Ib*, *sul2*, *dfrA14*, *tet*(A)

0

17-AB00587

S1-PFGE size (bp)	Unicycler-NextSeq	Flye-PacBio	Flye-ONT	Unicycler-PacBio/ NextSeq	Unicycler-ONT/ NextSeq
**30,000**	-<br>-<br>-	-<br>-<br>-	-<br>-<br>-	-<br>-<br>-	-<br>-<br>-
**100,000**	-<br>-<br>-	109,877<br>IncI1_α<br>*qnrS1*, *aad*A2, *lnu*(F)	109,622<br>IncI1_α<br>*qnrS1*, *aad*A2, *lnu*(F)	109,877<br>IncI1_α<br>*qnrS1*, *aad*A2, *lnu*(F)	109,876<br>IncI1_α<br>*qnrS1*, *aad*A2, *lnu*(F)
**150,000**	-<br>-<br>-	119,064<br>IncFIB(pHCM2) pHCM2<br>*bla*$_{CTX-M-1}$, *mph*(A)	118,872<br>IncFIB(pHCM2) pHCM2<br>*bla*$_{CTX-M-1}$, *mph*(A)	119,064<br>IncFIB(pHCM2) pHCM2<br>*bla*$_{CTX-M-1}$, *mph*(A)	119,064<br>IncFIB(pHCM2) pHCM2<br>*bla*$_{CTX-M-1}$, *mph*(A)

17-AB00639

S1-PFGE size (bp)	Unicycler-NextSeq	Flye-PacBio	Flye-ONT	Unicycler-PacBio/ NextSeq	Unicycler-ONT/ NextSeq
**< 20,000**	1,552<br>Col(MG828)<br>-	-<br>-<br>-	-<br>-<br>-	1,552<br>Col(MG828)<br>-	1552<br>Col(MG828)<br>-
	1,748<br>ColpVC<br>-	-<br>-<br>-		1,748<br>ColpVC<br>-	1,748<br>ColpVC<br>-
	-<br>-<br>-	-<br>-<br>-	6,719<br>-<br>-	-<br>-<br>-	3,374<br>-<br>-
	-<br>-<br>-	-<br>-<br>-	9,155<br>ColRNAI, ColRNAI<br>-	-<br>-<br>-	4,593<br>ColRNAI<br>-
**45,000**	-<br>-<br>-	47,133<br>IncX3, IncX1<br>*qnrS1*, *bla*$_{TEM-1}$	46,996<br>IncX3, IncX1<br>*qnrS1*, *bla*$_{TEM-1}$	47,132<br>IncX1<br>*qnrS1*, *bla*$_{TEM-1}$	47,132<br>IncX1<br>*qnrS1*, *bla*$_{TEM-1}$
**95,000**	-<br>-<br>-	105,628<br>IncI1_α<br>*aph*(6)-Id, *aph*(3'')-Ib, *aph*(4)-Ia, *aac*(3)-IVa, *mph*(A), *bla*$_{CTX-M-1}$	105,360<br>IncI1_α<br>*aph*(3'')-Ib, *aph*(4)-Ia, *aac*(3)-IVa, *mph*(A), *bla*$_{CTX-M-1}$	105,722<br>IncI1_α<br>*aph*(6)-Id, *aph*(3'')-Ib, *aph*(4)-Ia, *aac*(3)-IVa, *mph*(A), *bla*$_{CTX-M-1}$	105,776<br>IncI1_α<br>*aph*(6)-Id, *aph*(3'')-Ib, *aph*(4)-Ia, *aac*(3)-IVa, *mph*(A), *bla*$_{CTX-M-1}$
**100,000**	-<br>-<br>-	110,214<br>-<br>-	110,075<br>-<br>-	110,214<br>-<br>-	110,214<br>-<br>-
**140,000**	-<br>-	150,391<br>IncFII, IncFIB (AP001918)<br>*sul*2, *aph*(3'')-Ib, *aph*(6)-Id, *dfr*A5, *bla*$_{TEM-1}$	150,074<br>IncFII, IncFIB (AP001918)<br>*sul*2, *aph*(3'')-Ib, *aph*(6)-Id, *dfr*A5, *bla*$_{TEM-1}$	150,391<br>IncFII, IncFIB (AP001918)<br>*sul*2, *aph*(3'')-Ib, *aph*(6)-Id, *dfr*A5, *bla*$_{TEM-1}$	150,391<br>IncFII, IncFIB (AP001918)<br>*sul*2, *aph*(3'')-Ib, *aph*(6)-Id, *dfr*A5, *bla*$_{TEM-1}$

2

Supplement S5: Results of minimal inhibitory testing for tested *E. coli* isolates.

**Antibiotic**	**17-AB000050**		**17-AB000090**		**17-AB000432**		**17-AB000587**		**17-AB000639**	
**AMP**	**>64**	**NW**	**>64**	**NW**	**>64**	**NW**	**>64**	**NW**	**>64**	**NW**
AZI	4	WT	4	WT	8	WT	≤2 / 4/ 4	WT / WT / WT	4	WT
CEFEPI	0.5	NW	0.25 / ≤0.06/ 0.12	NW / WT / WT	32	NW	4/ 16/ 16	WT / NW / NW	32	NW
CHL	≤8	WT	≤8	WT	≤8	WT	≤8	WT	≤8	WT
CIP	0.5	NW	8	NW	>8	NW	0.25	NW	0.25	NW
COL	≤1	WT	≤1	WT	≤1	WT	2	WT	≤1	WT
ERTA	≤0.015	WT	≤0.015	WT	0.03	WT	≤0.015	WT	≤0.015	WT
FOT	8	NW	≤0.25	WT	>4	NW	>4	NW	>64	NW
FOX	8	WT	4	WT	4	WT	4	WT	4	WT
GEN	16	NW	1	WT	≤0.5	WT	1	WT	32	NW
IMIPE	0.25	WT	0.25	WT	0.25	WT	0.25	WT	0.25	WT
MERO	≤0.03	WT	≤0.03	WT	≤0.03	WT	≤0.03	WT	≤0.03	WT
NAL	8	WT	>128	NW	>128	NW	8	WT	8	WT
SMX	>1024	NW	≤8	WT	>1024	NW	≤8	WT	>1024	NW
TAZ	16	NW	≤0.5	WT	>8	NW	1	NW	1	NW
TET	≤2	WT	>64	NW	>64	NW	≤2	WT	≤2	WT
TGC	≤0.25	WT	≤0.25	WT	≤0.25 / 0.5/ ≤0.25	WT / WT / WT	≤0.25	WT	≤0.25	WT
TMP	≤0.25	WT	≤0.25	WT	>32	NW	≤0.25	WT	>32	NW

Isolates were tested in triplicate. Divergent outcomes are shown in triplicate. Minimal inhibitory concentrations were provided in mg/l. WT: wild-type, NW: non wild-type, represented in bold letters. Abbreviations: AMP: ampicillin, AZI: azithromycin, CEFEPI: cefepime, FOT: cefotaxime, FOX: cefoxitin, TAZ: ceftazidime, CHL: chloramphenicol, CIP: ciprofloxacin, COL: colistin, ERTA: ertapenem, GEN: gentamicin, IMIPE: imipenem, MERO: meropenem, NAL: nalidixic acid, SMX: sulfamethoxazole, TET: tetracycline, TGC: tigecycline, TMP: trimethoprim.

Supplement S6: Plasmid annotation of extrachromosomal elements carrying *qnrS1*. Sheet 1: pEC00050-17_5, sheet 2: pEC00090-17_2, sheet 3: pEC00432-17_3, sheet 4: pEC00587-17_1, sheet 5: pEC00639-17_4.

Supplement S7: Genome assemblies of the different WGS approaches.

References

1. Jolley, K.A. and M.C. Maiden, *BIGSdb: Scalable analysis of bacterial genome variation at the population level.* BMC Bioinformatics, 2010. **11**: p. 595.
2. Seemann, T., *abricate*. 2014: https://github.com/tseemann/abricate
3. Carattoli, A., et al., *In silico detection and typing of plasmids using PlasmidFinder and plasmid multilocus sequence typing.* Antimicrob Agents Chemother, 2014. **58**(7): p. 3895-903.
4. Coordinators, N.R., *Database resources of the National Center for Biotechnology Information.* Nucleic Acids Res, 2018. **46**(D1): p. D8-D13.
5. Feldgarden, M., et al., *Validating the AMRFinder Tool and Resistance Gene Database by Using Antimicrobial Resistance Genotype-Phenotype Correlations in a Collection of Isolates.* 2019.
